# Risk of symptomatic heterotopic ossification following plate osteosynthesis in multiple trauma patients: an analysis in a level-1 trauma centre

**DOI:** 10.1186/1757-7241-17-55

**Published:** 2009-10-13

**Authors:** Christian Zeckey, Frank Hildebrand, Philipp Mommsen, Julia Schumann, Michael Frink, Hans-Christoph Pape, Christian Krettek, Christian Probst

**Affiliations:** 1 Trauma Department, Hannover Medical School, Carl-Neuberg-Str.1, 30625 Hannover, Germany; 2 Trauma Department, University Hospital Aachen, Pauwelsstraße 30, 52074 Aachen, Germany

## Abstract

**Background:**

Symptomatic heterotopic ossification (HO) in multiple trauma patients may lead to follow up surgery, furthermore the long-term outcome can be restricted. Knowledge of the effect of surgical treatment on formation of symptomatic heterotopic ossification in polytrauma is sparse. Therefore, we test the effects of surgical treatment (plate osteosynthesis or intramedullary nailing) on the formation of heterotopic ossification in the multiple trauma patient.

**Methods:**

We retrospectively analysed prospectively documented data of blunt multiple trauma patients with long bone fractures which were treated at our level-1 trauma centre between 1997 and 2005. Patients were distributed to 2 groups: Patients treated by intramedullary nails (group IMN) or plate osteosynthesis (group PLATE) were compared. The expression and extension of symptomatic heterotopic ossifications on 3-6 months follow-up x-rays in antero-posterior (ap) and lateral views were classified radiologically and the maximum expansion was measured in millimeter (mm). Additionally, ventilation time, prophylactic medication like indomethacine and incidence and correlation of head injuries were analysed.

**Results:**

101 patients were included in our study, 79 men and 22 women. The fractures were treated by intramedullary nails (group IMN n = 50) or plate osteosynthesis (group PLATE n = 51). Significantly higher radiologic ossification classes were detected in group PLATE (2.9 ± 1.3) as compared to IMN (2.2 ± 1.1; p = 0.013). HO size in mm ap and lateral showed a tendency towards larger HOs in the PLATE group. Additionally PLATE group showed a higher rate of articular fractures (63% vs. 28% in IMN) while IMN demonstrated a higher rate of diaphyseal fractures (72% vs. 37% in PLATE; p = 0.003). Ventilation time, indomethacine and incidence of head injuries showed no significant difference between groups.

**Conclusion:**

Fracture care with plate osteosynthesis in polytrauma patients is associated with larger formations of symptomatic heterotopic ossifications (HO) while intramedullary nailing was associated with a higher rate of remote HO. For future fracture care of multiply injured patients these facts may be considered by the responsible surgeon.

## Background

Heterotopic ossification (HO) after trauma still remains poorly understood. Hormonal as well as systemic and external factors are discussed to induce the HO [1-5]. Heterotopic ossification is described as a result of the inappropriate differentiation of pluripotential mesenchymal cells into osteoblastic cells influenced by local and systemic factors such as local presence of bone morphogenetic protein (BMP) or increased systemic expression of prostaglandine-E2 [6]. The newly formed bone has been found biologically highly active with high formation rates and high osteoclastic density [7].

Furthermore, this process is a systematic progression from osteoid to calcification within weeks and is mostly seen around the hip after internal fracture stabilisation or total hip arthroplasty [6]. Further studies showed the highest incidence of HO at the hip joints, followed by the knee [8], elbow [9] and shoulder [10]. Widely accepted complications due to HO are persistent pain and functional limitations [6]. Additionally, ankylosis is a well known problem and occurs in up to 25% of the patients [3,11,12].

Risk factors to sustain HO were classified by Ellerien in three main groups of individual injury, personal and therapeutic factors [13]. Subsequently several studies revealed the occurrence of HO in patients with severe head injury [14-16]. Furthermore, prolonged ventilation time is accepted as a contributing factor.

Since treatment of HO oftentimes is difficult and recurrence rates are high, prevention of HO became increasingly important [6]. As medical treatment, protective effects of indomethacine or selective cyclooxygenase (COX)-2 inhibitors could be shown [17-19].

However, besides the effects of head injury and mechanical ventilation, little is known about HO formation in acute trauma patients following operative fracture care treatment. Therefore we studied, if type of surgical fracture care affects HO formation in polytrauma patients.

## Methods

The study followed the guidelines of the revised UN declaration of Helsinki in 1975 and its latest amendment of 1996 (42nd general meeting). The population of our study includes 101 polytrauma patients with fractures of the long bones of either upper or lower extremity which were treated at our level-1 trauma centre between 1997 and 2005. Inclusion criteria were detected HO on x-rays (2 views) 3-6 months after trauma, 3-6 months follow-up, age between 16-65 years and ISS ≥ 16. Exclusion criteria were HO after arthroplasty, surgical treated spinal fractures as well as fractures of the ankle, foot, wrist and hand.

Patients were distributed to the following groups:

1.) Multiple trauma patient treated by intramedullary nails (group IMS)

2.) Multiple trauma patient treated by plate osteosynthesis (group PLATE)

### Scoring systems

To reveal trauma severity, the Injury Severity Score (ISS) [20,21] and the Abbreviated Injury Scale (AIS) [22] were used. The presence or absence of a head injury was classified by initial GCS and simultaneous CT-Scan abnormalities such as fractures of the skull or intra-cranial injuries. Patients with an almost normal to normal GCS and combined anatomical lesions on the CT-scan were classified as head injured patients.

### Analysis of the HO - clinical and diagnostic assessment

**Figure 1:**
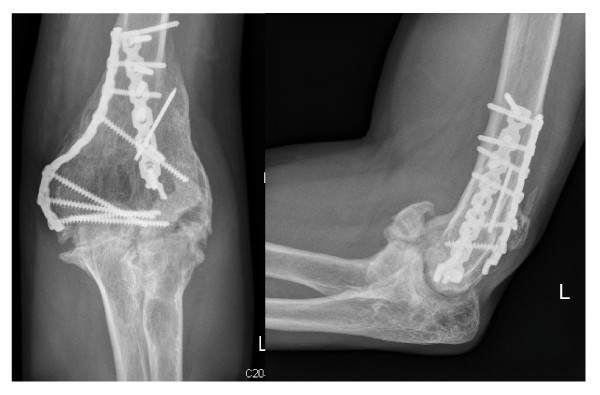
**Heterotopic ossification following plate osteosynthesis of a distal humerus fracture**.

**Figure 2:**
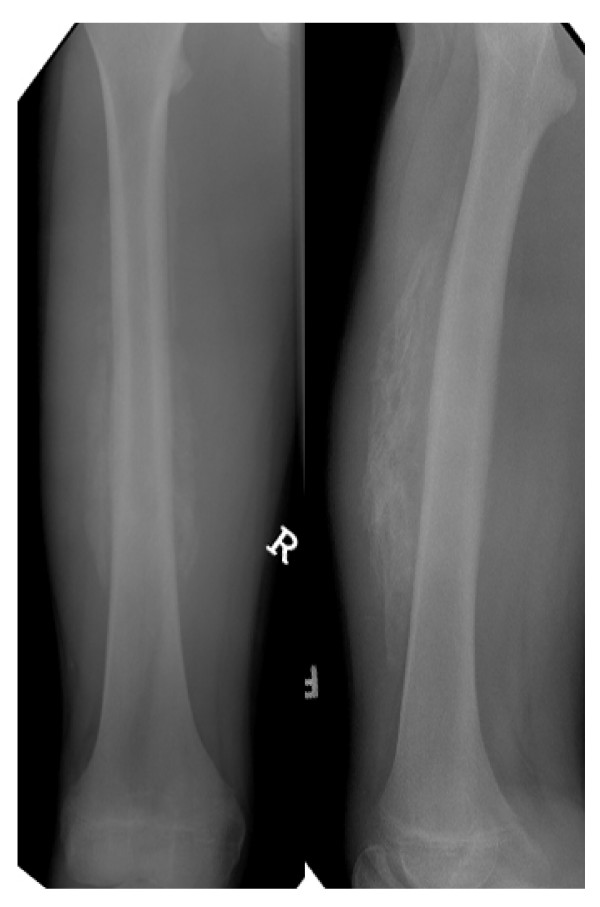
**Heterotopic ossification remote to the fracture site at the contralateral femur**.

Patients with symptomatic HO at routine follow-up in our clinic were included in the present study. A great part of heterotopic ossifications cause swelling, pain or limited function to total ankylosis. Since these patients confront the clinician during every day work and utilize clinical resources, we focussed on these patients. We asked and examined the patients towards one ore more of these symptoms and took x-rays of the affected body region in standardized antero-posterior and lateral views from the follow-up appointment three to six months after the initial injury for radiologic confirmation of suspected HO (figure 1, figure 2).

Today, Brooker's classification is widely accepted for classification of the HO around the hip joints, classifying HO into 4 grades ranging from just visible (grade 1) to total ankylosis (grade 4) in standardized x-rays in two planes [23]. Unfortunately, a general and comparable classification system of all joints to date does not exist. We therefore adapted and modified Brooker's classification in a similar way to the other joints and defined the extent of the heterotopic ossification accordingly (grade 1-grade 4, in the following "radiologic ossification class"). Additionally, the maximum expansion on both films was measured in mm. Furthermore, the location at the fracture site (fractured long bone between the adjacent joints) or at a site remote to the fracture site (any non-adjacent part of an extremity) was noted. All the x-rays were analysed and classified by two independent trauma surgeons (J. S. and C. P.).

### Pharmacotherapy

Patients were defined to receive prophylactic medications, if corticoids, non-steroidal anti-inflammatory drugs (NSAIDs), muscle relaxants, diphosphonates or hyaluronidases were administered in a prophylactic regimen.

### Operative treatment

We defined surgical fracture if initially intramedullary nailing, plate osteosynthesis or external fixateurs with secondary conversion to intramedullary nailing (damage control orthopaedics, DCO) were used. No other methods of fracture care such as extension treatment or casting were used in our population.

### Intensive care treatment

Ventilation time and duration of intensive care unit stay were analysed.

### Statistics

Results are shown as mean ± standard error of the mean (SEM). For the analysis of nominal-scaled variables the Chi-squared test (Chi^2^) was used, for continuous data we used the student t-test. In addition, analysis of variances (ANOVA) was performed followed by post-hoc Tukey test to determine differences between groups. Level of significance was set at p < 0.05.

## Results

### Demographic data

The study population consisted of 79 men (78.7%) and 22 women (21.3%). Average age between groups showed no significant difference (IMN: 27.1 ± 3.1 vs. PLATE 29.1 ± 2.6 years, p = 0.25). The GCS mean value was also statistically comparable between groups (IMN: 10.7 ± 0.8; PLATE 11.0 ± 1.0; p = 0.93) as was the incidence of head injuries (IMN: 33% vs. PLATE: 24%; p = 0.36).

Additionally, PLATE group showed a higher rate of articular fractures (63% vs. 28% in IMN; p = 0.003) while IMN demonstrated a higher rate of diaphyseal fractures (72% vs. 37% in PLATE; p = 0.003).

Comparing the mean ISS, and AIS max there was no statistical difference between our groups (table 1).

**Table 1:** AIS and ISS-values for the groups without significant differences.

	**IMN**	**PLATE**
AIS head	3.3 ± 2.1	3.0 ± 1.4

AIS face/neck	1.6 ± 0.7	1.5 ± 0.6

AIS spine	3.7 ± 2.3	4.1 ± 2.0

AIS thorax	4.1 ± 2.8	3.6 ± 2.7

AIS abdomen	1.8 ± 0.7	2.1 ± 0.9

AIS upper extremity	2.2 ± 0.9	1.9 ± 0.7

AIS lower extremity	2.6 ± 0.8	2.3 ± 0.7

		

AIS max	4.6 ± 2.2	4.4 ± 1.9

ISS	44.3 ± 27.4	42.1 ± 25.0

### Incidence, size and localisation of HO

**Figure 3:**
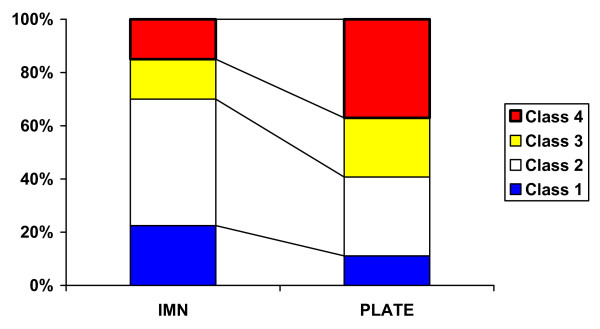
**Percentage of patients with respective radiologic classes**. PLATE patients showing significantly more Brooker values of 3 and 4.

A significantly higher incidence of radiologic classes 3 or 4 was found for the PLATE group in comparison to the IMN group (p = 0.04; figure 3).

For the largest extension of the HO in mm in two views of plane x-rays the p-values show no significant difference but a tendency towards larger HO-formations in group PLATE (table 2).

**Table 2:** Expression of the HO

	**IMN**	**PLATE**	
a.p. (mm)	21 ± 2	26 ± 3	0.1337

lat. (mm)	16 ± 2	22 ± 5	0.1092

**Figure 4:**
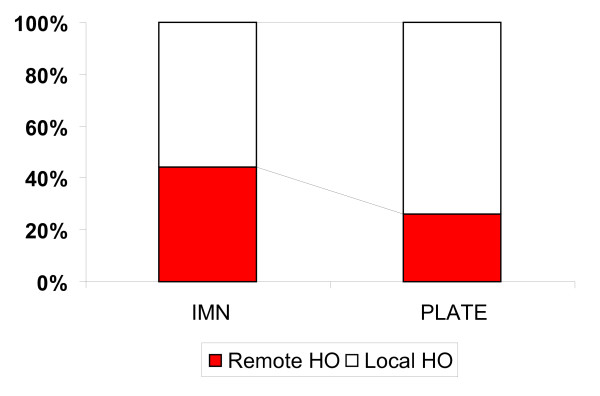
**Percentages of remote and local HO**. Significantly more remote HO in the IMN group compared to the PLATE group.

HO occurred significantly more frequently remote to the fracture site in the IMN group in comparison to the PLATE group (p = 0.03; figure 4).

### Effect of ICU and medical treatment

No differences of ventilation time (IMN: 12.2 ± 3.1 days vs. PLATE: 11.0 ± 2.7 days; p = 0.48), duration of the ICU-stay (IMN: 14.6 ± 3.9 days vs. PLATE: 13.2 ± 3.6 days; p = 0.76) and indomethacine prescribed (IMN: 22% vs. PLATE: 30%; p = 0.47) was demonstrated.

## Discussion

The formation of HO in trauma patients is critically discussed in the context of fracture healing. The role of severe head trauma was described in former studies [1,14,15,24]. Studies on the influence of multiple trauma in combination with severe head trauma were performed in our department [5,7] and confirmed the role of head injuries in polytrauma, too. In the present setting, we addressed the question of the impact of the applied surgical therapy of long bone fractures in polytrauma patients on the development of symptomatic HO. In the present setting, we specifically focussed on symptomatic HO. This is important due to the fact that only these patients are suffering from the HO. The patients included in our study are representative for patients suffering from the complaints following major trauma. The need for diagnostic and sometimes therapeutic interventions in these patients is crucial and towards symptomatic HO difficult. Therefore, we could not demonstrate an over-all incidence of heterotopic ossification. In our understanding, inappearant HO should not be treated and are to categorize as diagnostic findings by chance.

The present study is a retrospective single centre analysis of prospectively collected patient data. Demographic and injury related data of our patients are similar to those published before: Multiply injured patients commonly group around the age of 30 to 40 years with a predominance of males as do our patients. Overall injury severity and injury pattern are consistent with other cohorts [25]. Similarly, the GCS of our patients is comparable to data of other authors [26,27].

Furthermore, good comparison of patient groups seems possible because treatment strategy was very consistent in our centre over the inclusion period. Required data were documented completely for all of the individuals. Two independent examiners of the x-rays lead to similar results. Overall, we feel that our analysis safely leads to the following results:

• In polytrauma patients, plate osteosynthesis is followed by larger HO formations compared to intramedullary nailing.

• Patients treated with intramedullary nails more commonly showed HO formations remote to the fracture site.

Nonetheless, there are some limitations to our study. Heterotopic ossifications were essentially described by Brooker et al. This classification system includes the HO around the hip joint and is now widely accepted for classification following acetabular fracture treatment and arthroplasty of the hip. To classify the functional status of the hip joint, the Harris score is widely known. Further classifications were developed for the elbow, this score is divided into radiologic and functional aspects [28]. Since there is no general classification system for all the joints, we transferred the Brooker criteria for the four different classes accordingly to the large joints of the extremtities.

### Effects of injury pattern

The role of head injuries in the formation of HO still is lively debated about in the literature. Some authors reported a stimulation of fracture healing in patients with head injuries [29-31]. Furthermore, a positive correlation of the severity of the head injury and the HO rate was observed [24]. Other studies could not confirm a relationship between severe head trauma and HO formation. Lehmann et al. demonstrated constant expressions of the HO in multiply injured patients without head trauma in comparison to multiply injured patients with severe head trauma [4]. We could confirm the findings of Lehmann et al., the present report could demonstrate comparable GCS and constant incidence of head trauma in both groups.

Interestingly, a recent study demonstrated differences in the location of the HO between polytrauma patients with and without severe head trauma. In polytrauma patients with associated head trauma, the HO was located adjacent to the fracture region. In polytrauma patients without head injury, the HO formation more frequently occurred at sites remote to the actual fracture sites [7]. In our study, the incidence and severity of head injuries was distributed equally between both groups.

Nonetheless, we found a higher incidence of remote HO in the IMN group, leading to the idea of systemic factors liberated during nailing that affect HO formation such as prostaglandin E2 [1,3,32].

### Effects of treatment strategy

Surgical treatment such as osteosynthesis, manipulation at joints or traumatic haematoma is known to be a risk factor for the development of the HO [6,33,34]. In the present study, we could demonstrate a positive association of plate osteosynthesis and the development of the HO in the PLATE group.

A more invasive approach required for plate osteosynthesis is well described as one of the risk factors [6]. Local fracture and soft tissue manipulation is believed to hold a substantial role in the development of the HO, possibly by the liberation of bone morphogenetic protein (BMP) or other tissue factors [35,36]. Home et al reported on extended HO after intramedullary nailing in combination with severe head trauma [37]. However, these results could not be shown in our study potentially due to a relatively low patient number.

### Effects of additional therapy

In the present study, there were no significant differences in ventilation time (IMN: 12.2 ± 3.1 days vs. PLATE: 11.0 ± 2.7 days; p = 0.48). Long term ventilation is widely accepted as a factor associated with HO formation [2]: One study showed HO in patients after pulmonary transplantation with prolonged ventilation times at healthy joints [38]. Mechanical ventilation may lead to changes in the acid-base metabolism which results in mineral accumulation in the soft tissues and therefore may lead to HO formation [5] which was also demonstrated in an experimental study [34]. Other authors speculate that HO formation in shock trauma patients and mechanically ventilated patients occurs due to critical hypoxia in consequence to local tissue compression. It could be revealed that osteogenesis is induced by low oxygen concentrations [33].

### Effects of prophylactic medication

Prophylactic medications to prevent or to decrease HO are widely discussed in hip and acetabular surgery. Moreover, several studies revealed the effectiveness of prophylactic treatment after knee arthroplasty [18,19,39]. Prophylactic strategies may lead to decrease the development and the resulting size of the HO; these strategies include treatment with NSAID or postoperative radiotherapy. Best evidence for prophylactic medication is shown for indomethacine for at least 7 days, other NSAIDs are also well documented [19]. To our knowledge, there are no reports on the effect of prophylactic medication on HO formation in multiple trauma patients. In our study, up to 30% (group PLATE) of the patients received prophylactic medications, there were no differences of NSAIDs prescribed (IMN: 22% vs. PLATE: 30%; p = 0.47).

The missing effect of the prophylactic treatment in our study may be the result of the low fraction of patients who received prophylactic treatment. On the other hand, HO formation in multiply injured patients may result out of interactions of multiple systemic and local factors, thereby limiting the effect of a single intervention or substance.

## Conclusion

We demonstrate that fracture care by plate osteosynthesis in multiple trauma patients is significantly associated with the formation of symptomatic heterotopic ossifications. We also found intramedullary nails being associated with a higher incidence of HO remote to the fracture site. Since HO was shown to lead to considerable long term complaints, our results may serve clinicians to critically verify their strategies for acute fracture care in multiple trauma patients to prevent future HO formation. However, the individual therapeutic approach has to be subject to the patient's status.

## Competing interests

Financial competing interests:

The author(s) declare that they have no competing interests

Non-financial competing interests:

There are no non-financial competing interests (political, personal, religious, ideological, academic, intellectual, commercial or any other) to declare in relation to this manuscript.

## Authors' contributions

CZ performed data analysis and interpretation and drafted the manuscript. FH interpreted data and helped drafting the manuscript. PM carried out data analysis. JS has made substantial contributions to acquisition of data. MF participated in data analysis and interpretation. HCP made substantial contributions to conception and design of the study. CK made substantial contributions to conception of the study. CP performed statistical analysis and helped to draft the manuscript. All authors read and approved the final manuscript.
